# Cardiac Arrest Due to Pulmonary Embolism Without Clinical Features of Deep Vein Thrombosis in a Young Female: A Rare Presentation of May-Thurner Syndrome

**DOI:** 10.7759/cureus.68596

**Published:** 2024-09-04

**Authors:** Yagnya D Dalal, Devagna P Mehta, Kelly Alford

**Affiliations:** 1 Internal Medicine, Gujarat Cancer Society Medical College, Hospital and Research Centre, Ahmedabad, IND; 2 Nephrology, Research Medical Center, Kansas City, USA; 3 Infectious Diseases, University of Miami Miller School of Medicine, Miami, USA

**Keywords:** venous thromboembolism (vte), iliac vein compression, deep vein thrombosis (dvt), may thurner syndrome (mts), pulmonary emboli

## Abstract

Deep vein thrombosis (DVT) is a serious condition with a high disease burden. Pulmonary embolism is one of the disastrous complications of DVT. The etiology of DVT includes factors responsible for hypercoagulation, venous injury, and factors causing stasis in the deep veins. May-Thurner syndrome (MTS) is one of the rarely thought of causes of DVT. MTS is an anatomical variant where the right common iliac artery compresses the left common iliac vein against the lumbar vertebrae. This leads to thrombus formation and stenosis in the left common iliac vein at the site of cross-over, resulting in an iliofemoral DVT. We present a case of a young female who presented with acute bilateral pulmonary embolism and subsequent cardiac arrest. She was treated with mechanical thrombectomy, angioplasty, and stent placement under the umbrella of anticoagulant agents. We aim to present this case to highlight that MTS should be considered a differential etiological condition in iliofemoral DVT. MTS is a rarely considered condition by clinicians while evaluating patients with lower limb DVT. When unaddressed, MTS can lead to recurrent DVT, post-thrombotic syndrome, and fatal complications like pulmonary embolism. Clinicians should investigate for possible MTS in patients with left lower extremity venous thrombotic events, irrespective of the presence of other risk factors.

## Introduction

May-Thurner syndrome (MTS), first described by May and Thurner in 1957, is an anatomical variant where the right common iliac artery (RCIA) compresses the left common iliac vein (LCIV) against the fifth lumbar vertebra [1,2]. MTS is also referred to as iliocaval venous compression syndrome or Cockett’s syndrome [3]. The direct compression of the LCIV by the RCIA causes irritation of the endothelial surface due to the pulsatility of the overlying artery and thus induces intimal proliferation in LCIV [4,5]. Direct compression and endothelial proliferation lead to a narrowing of the lumen of the LCIV. This condition, therefore, causes venous outflow obstruction at the site of cross-over and a subsequent increased risk of developing deep vein thrombosis (DVT) in the ipsilateral lower limb. Recent studies have found that the prevalence of MTS in the general population is around 24% [6]. However, out of all the cases of lower limb DVTs, MTS has been reported in only 2-3% [7]. Although most of the patients with MTS are asymptomatic, the most common presentation is DVT [2]. Moudgill et al. reported that out of 113 patients they reviewed, 77% of the patients presented with DVT [2]. Though rare, cases of right-sided venous thromboembolism are also reported [3,5]. The majority of DVTs can satisfactorily be treated with anticoagulation therapy. However, in patients with underlying MTS, there is an increased risk of recurrent thromboembolic events, which mandates the need for endovascular procedures [3]. We report a case of a young female who experienced cardiac arrest due to an unprovoked pulmonary embolism (PE). Our patient had no traditional risk factors for DVT or PE. PE occurring in the absence of signs of DVT in the context of MTS is exceedingly rare. This case illustrates the importance of considering anatomical and potentially underdiagnosed conditions like MTS in patients with atypical presentations and no apparent risk factors for thromboembolic events.

## Case presentation

**Figure 1:**
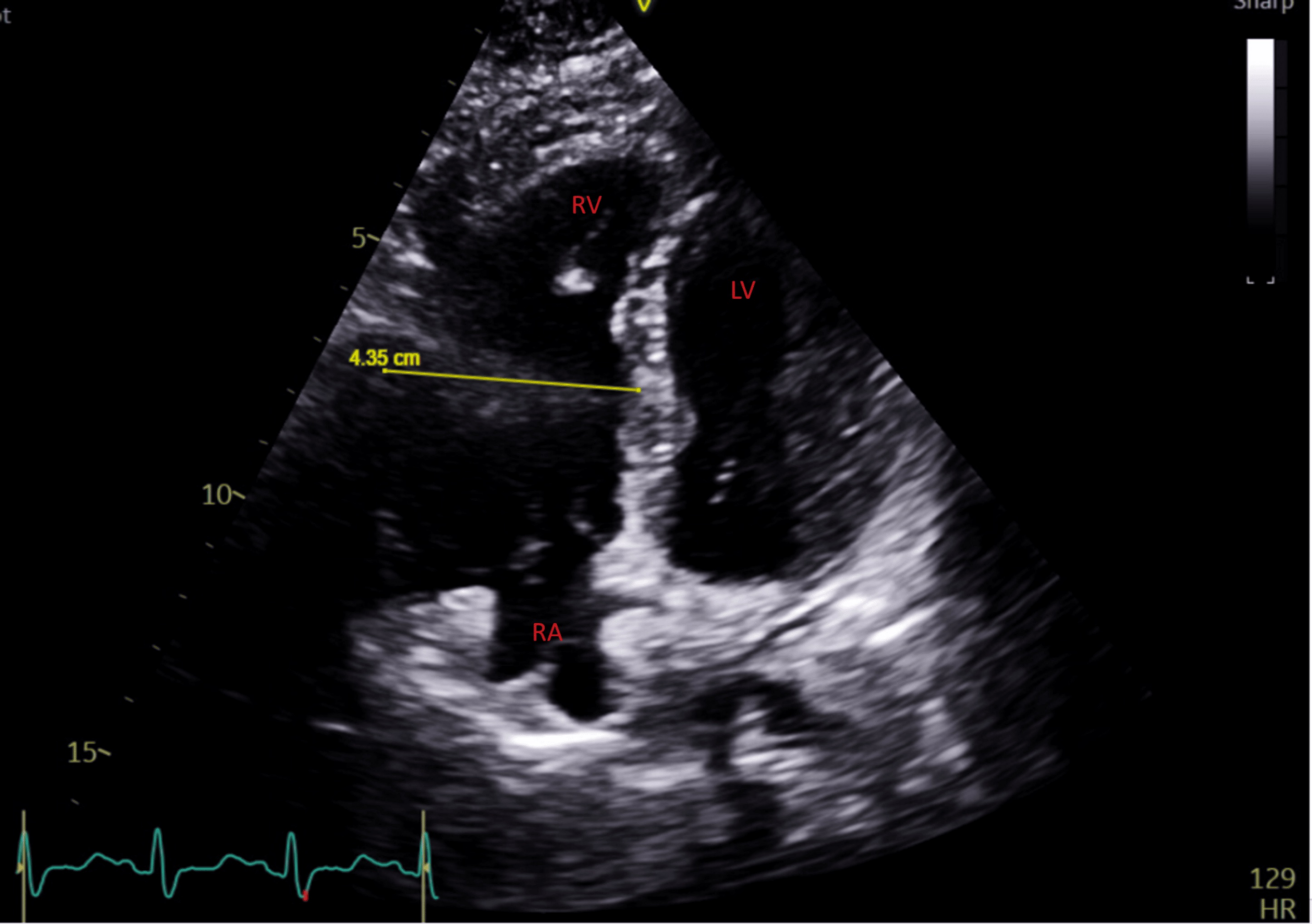
Significant right ventricular dilatation on echocardiography. The mid-diameter of the right ventricle on echocardiography measured 4.35 cm, which is above the cutoff value of 3.5 cm.

A 25-year-old woman was admitted to a psychiatric hospital for management of schizophrenia. She was not eating much at home and had lost 25 lbs over the past four weeks. She was started on olanzapine. During the second night of hospitalization, she was noted to be ill. Pulse oximetry showed new onset hypoxia with oxygen saturation of 70%. Shortly after, she collapsed and was noted to be without a pulse. She was brought to the emergency room with the CPR in progress. Advanced cardiac life support (ACLS) protocol was continued due to pulseless electrical activity cardiac arrest. Return of spontaneous circulation was achieved 30 minutes later. Shortly after that, the patient had another brief episode of cardiac arrest. Bedside ultrasonography showed the presence of a significantly dilated right ventricle (Figure 1). The mid-diameter of the right ventricle on echocardiography measured 4.35 cm, which is above the cutoff value of 3.5 cm.

**Figure 2:**
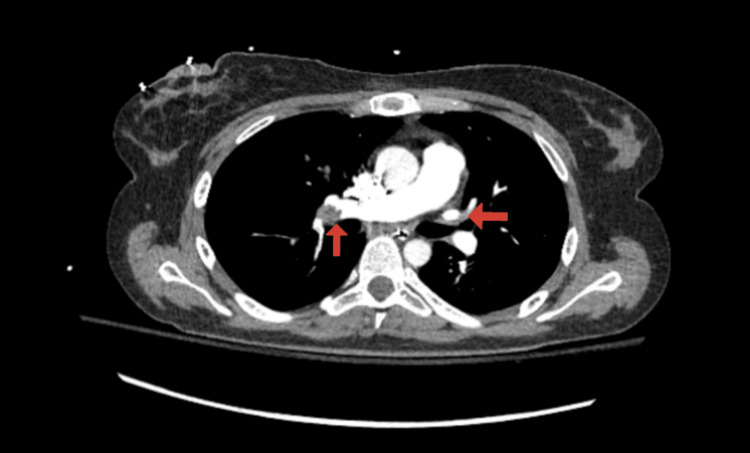
Red arrows showing right-sided predominant bilateral pulmonary embolism.

**Figure 3:**
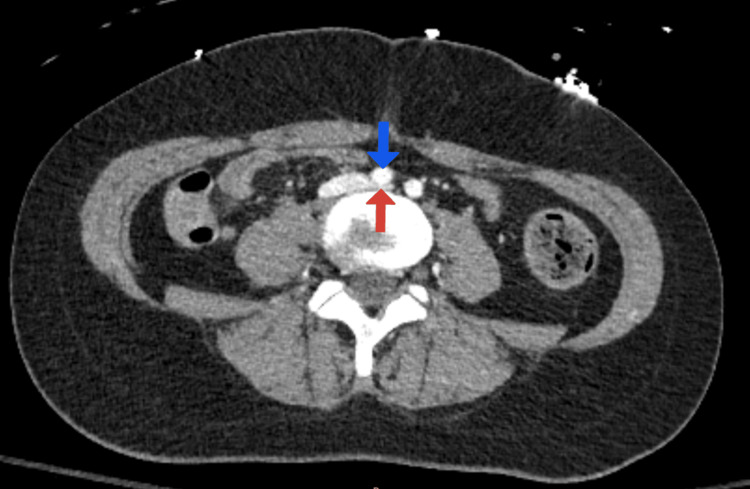
Compression of the left common iliac vein (red arrow) by the right common iliac artery (blue arrow) consistent with May-Thurner syndrome.

Tenecteplase 30 mg was administered once intravenously under the working diagnosis of PE. Return of spontaneous circulation was obtained again. The chest CT scan showed the presence of right-sided predominant bilateral PE (Figure 2). PE thrombectomy was done using the INARI FlowTriever (Inari Medical, Irvine, CA) device, and an infrarenal retrievable inferior vena cava (IVC) filter was placed. The left pelvic venogram showed partially occlusive LCIV thrombosis. A prior CT scan of the abdomen and pelvis showed the RCIA compressing over the LCIV, which was consistent with MTS (Figure 3).

The past medical history is consistent with depression, schizophrenia, and panic attacks. She did not have complaints of lower extremity swelling or pain. There was no previous history of DVT. She had no relevant family or surgical history. She was intubated and was put on a ventilator with FiO_2_ of 100%, positive end-expiratory pressure (PEEP) of 5 cm of H_2_O, respiratory rate of 18/minute, and tidal volume of 450 mL. Weight-based continuous heparin infusion was started. She did not have lower extremity edema, induration, or limb-size discrepancy. The hematological investigations were negative for the hypercoagulable state (Table 1).

**Table 1:** Results of important investigations INR - international normalized ratio, aPTT - activated partial thromboplastin time

Test	Patient value	Reference value
Prothrombin time (seconds)	18.7	9.4-12.5
INR	1.7	0.9-1.1
aPTT (seconds)	59.3	25.1-36.5
Viral panel	Negative	-
Urine drug screen	Negative	-
Alcohol (mg/dL)	<10	<10

She developed thrombocytopenia, requiring a change from heparin to Argatroban. She was extubated on the fourth day of the admission.

**Figure 4:**
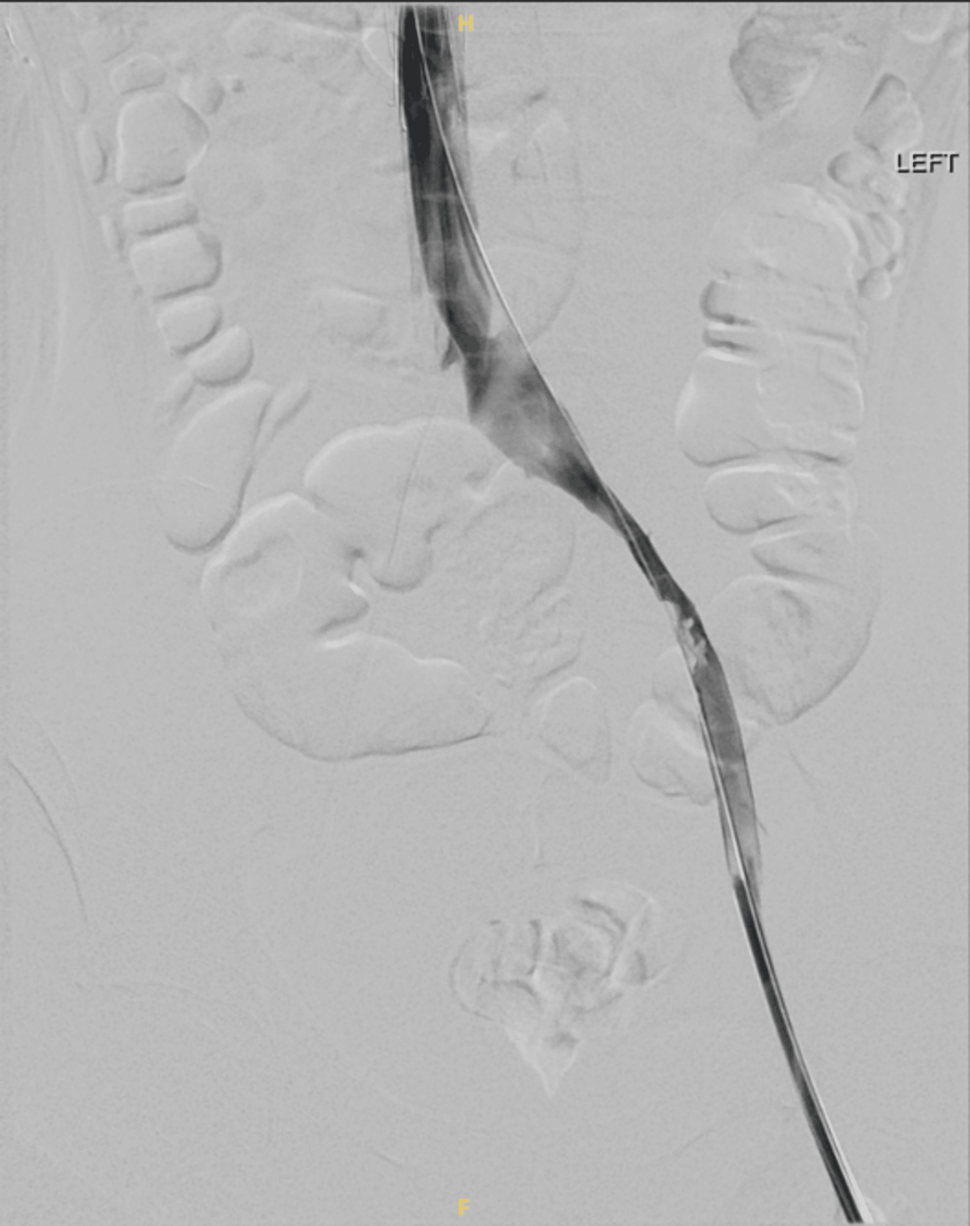
Non-occlusive thrombi in the left common and external iliac veins.

**Figure 5:**
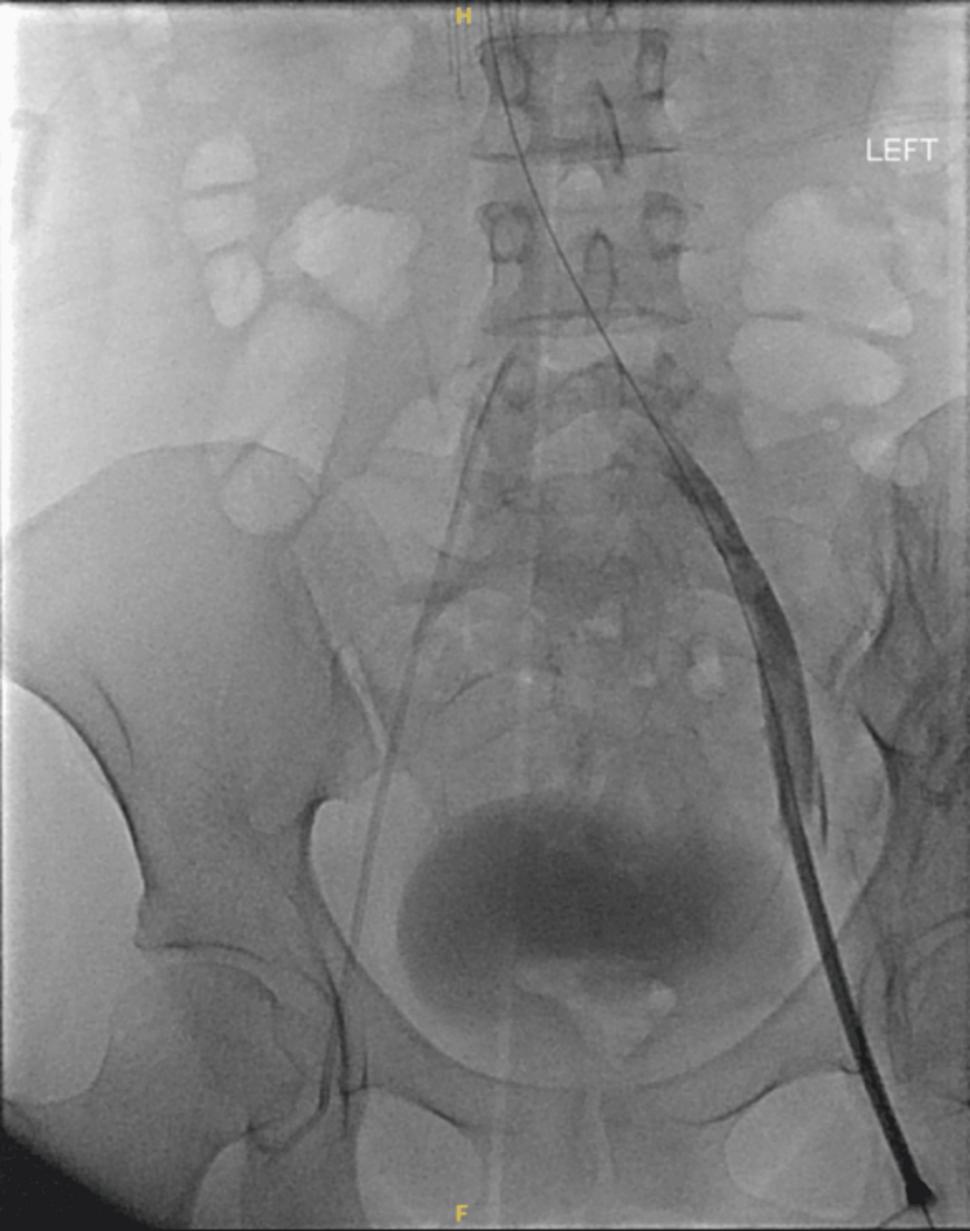
Angioplasty followed by stent placement.

**Figure 6:**
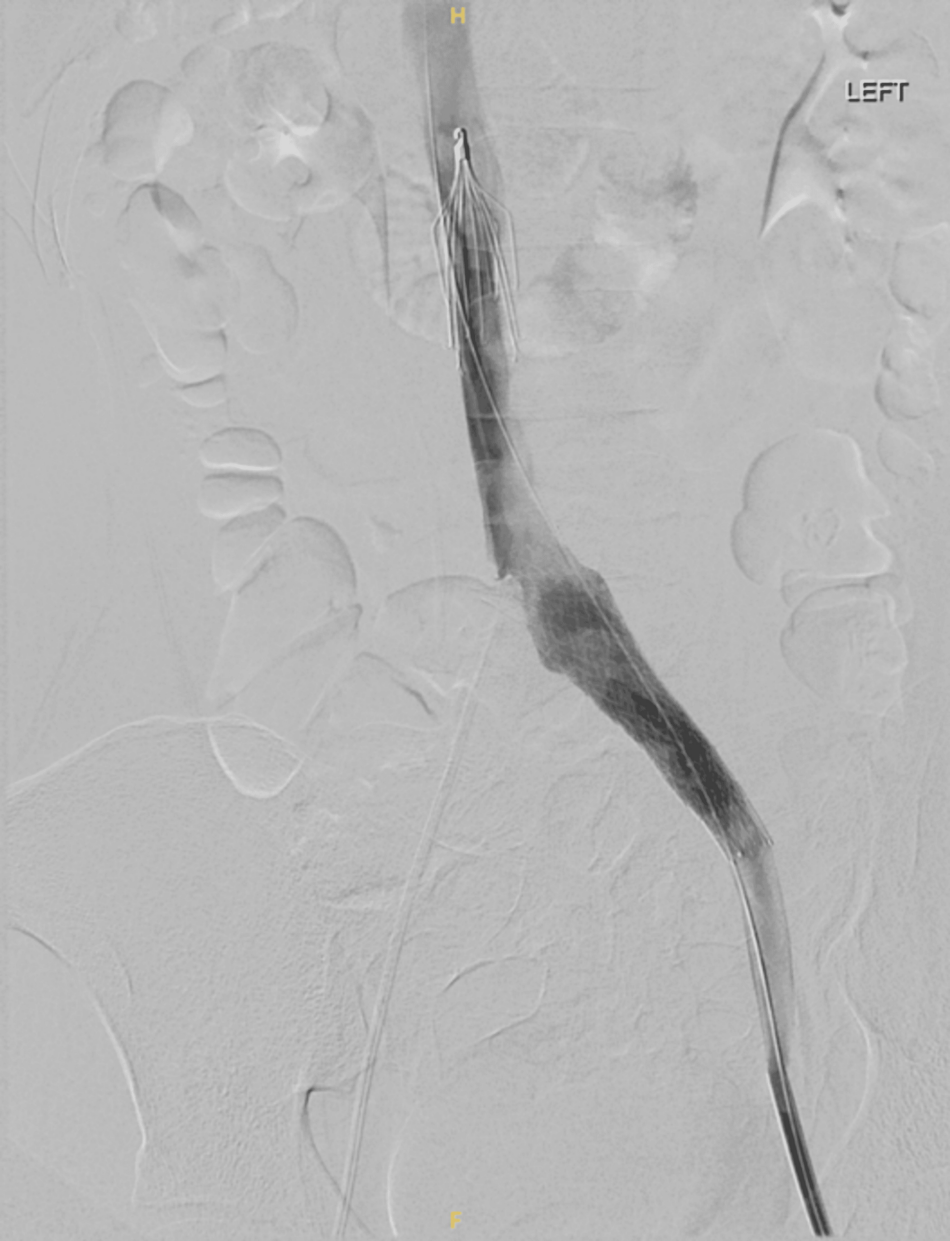
Good blood flow through the stent after the procedure. The image also shows the presence of a retrievable inferior vena cava (IVC) filter.

When the patient’s condition stabilized on the sixth day of the admission, CT venography was done. It showed the presence of flow-limiting external compression on the LCIV, long segment flow-limiting stenosis throughout the left common iliac and external iliac veins, and superimposed nonocclusive chronic eccentric thrombus throughout the left common and external iliac veins (Figure 4). The nonocclusive thrombi in the left common iliac and external iliac veins were treated via mechanical thrombectomy using 12 × 60 mm and 14 × 60 mm Atlas balloons. The superimposed long segment flow-limiting stenosis from the left common iliac to the external iliac vein was treated via prolonged angioplasty from the LCIV to the distal external iliac vein using 12 × 60 mm and 14 × 60 mm Atlas balloons. The flow-limiting external compression on the LCIV seen on recent CT was treated via stent placement from the origin of the LCIV to the left external iliac vein using a 16 × 80 mm Venovo stent (Figure 5). Overall, significantly improved luminal patency and flow throughout the LCIV and left external iliac vein were seen post-intervention (Figure 6).

The patient was discharged on oral Apixaban 5 mg twice daily and was advised to follow up in the outpatient setting.

## Discussion

MTS is named after May and Thurner, who, in 1957, discovered the changes in the endothelium of the LCIV in the area of its crossing over the RCIA and described them as “spurs” [8]. Though this condition was recognized a long time ago, it still remains a less-known problem with limited available literature. It is a common but rarely diagnosed condition that involves LCIV compression by the RCIA, causing venous outflow obstruction at the cross-over with resultant venous hypertension in the left lower limb. The suspected prevalence of this disorder is 24% in the general population in retrospective studies of computed tomography scans and 22% in cadaveric studies [3,6]. This condition should be suspected in cases of DVT, especially in those patients that meet certain known risk factors such as female gender, left lower extremity involvement, or recurrent episodes of lower limb DVT [7]. A common presentation of this condition is episodic lower extremity swelling, varicosities, venous ulcers, or stasis dermatitis [5]. Sometimes, this condition may present as a lower-limb DVT [5]. Venous stasis and hypertension caused by venous outflow obstruction in the iliocaval region are responsible for these presentations. Despite being a common condition, MTS is an uncommon cause of DVT, accounting for approximately 2-5% of lower-extremity venous disorders [9].

This case report is unique because it shows an unexpected and unusual presentation of MTS that has not been documented much in the literature. Our patient presented with PE without any evidence of DVT or chronic lower extremity venous insufficiency. It suggests that MTS is an underdiagnosed condition and highlights the importance of identifying these patients, as more invasive treatment is required to reduce the risk of chronic venous hypertension and its sequela, like chronic venous insufficiency, venous ulcer, etc. The risk factors for MTS include female gender, postpartum, multiparity, history of oral contraceptive consumption, scoliosis, dehydration, and hypercoagulable disorders [2-3,10]. Our patient’s risk factors were female gender and possible dehydration, as she lost 25 lbs in the last four weeks. Because of the overlap of risk factors between DVT and MTS, MTS is often overlooked. Many clinicians may attribute DVTs to these risk factors instead of this underlying anatomical pathology [9].

The approach to investigating the MTS varies among clinicians. Some authors recommend that patients with risk factors for MTS should be investigated [1,11]. However, MTS is noted even in young males without any risk factors [9]. Thus, an approach to the diagnosis of MTS should be a combination of clinical presentation, despite the risk factors, and imaging findings. Any patient presenting with lower limb swelling or pain should be investigated for DVT with MTS considered as a cause [10,12]. Duplex ultrasound should be performed as a first-line investigation because it is noninvasive and can assess dynamically for both DVT and MTS. However, variables, such as the patient's body habitus, the location of iliac veins deep in the pelvis, and the sonographer's experience, may limit the efficacy of duplex ultrasound. The visualization of the common iliac vein by duplex ultrasound was estimated to be only 47% [10,13]. Other imaging modalities include CT venography, MR venography, and conventional venography [14]. Contrast venography is considered the investigation of choice for MTS [4]. CT venography should be considered if duplex US does not adequately assess the LCIV. The timing of venous phase contrast in CT venography can be difficult as both inherent patient factors (such as cardiac output) and varying levels of venous obstruction can unpredictably affect contrast transit time, which can result in obtaining suboptimal images [15]. However, most of the authors consider CT venography the gold standard modality to detect MTS [4,11]. CT venography is also of benefit in detecting other causes of venous compression, such as a pelvic mass, and outlining the collateral pathways [12]. CT has a limited role in pregnancy due to radiation hazards, and it can overestimate the degree of compression in dehydrated patients [1].

In our patient, a CT with contrast study accurately diagnosed MTS. The management of MTS is aimed at the removal of the clot in the LCIV with pharmacological thrombolysis or mechanical thrombectomy to prevent post-thrombotic syndrome [16]. In patients with iliofemoral DVT due to MTS, endovascular thrombolysis with anticoagulants is superior to anticoagulation therapy alone [14]. Surgical management includes open procedures or more modern endovascular therapy with angioplasty and stenting, followed by anticoagulation [7]. Re-stenosis after stent placement can occur rarely [7]. The chances of post-stenting stenosis are higher in patients with concomitant atherosclerosis [7]. Prophylactic IVC filter placement before thrombolysis or thrombectomy is not recommended [14]. Unfractionated heparin carries a higher risk of hemorrhage, so low-molecular-weight heparin (LMWH) and factor Xa inhibitors (Rivaroxaban and Apixaban) are considered safe [17].

Our patient presented with bilateral PE with right-sided predominance. PE thrombectomy was done using the INARI FlowTriever device, and an infrarenal retrievable IVC filter was placed. She was initially put on a titrated heparin drip but subsequently switched to Argatroban secondary to thrombocytopenia. Once stabilized, she was treated with mechanical thrombectomy of the left common and external iliac veins, followed by angioplasty and stenting. Significant improvements in luminal patency and blood flow were noted after the intervention. She was put on oral Apixaban on discharge.

## Conclusions

MTS is less thought of and is, therefore, an underdiagnosed disorder. It should be considered in patients with left lower limb DVT, recurrent lower limb DVT, or signs of lower limb venous insufficiency. CT venography is a gold-standard diagnostic imaging technique. Anticoagulants alone are not sufficient to treat the DVT caused by MTS. Timely diagnosis and appropriate treatment with pharmaco-chemical thrombolysis or mechanical thrombectomy along with angioplasty with or without stenting would reduce the morbidity of post-thrombotic syndrome. When unaddressed, MTS can lead to recurrent DVT, post-thrombotic syndrome, and fatal complications like PE. Our case is unique as the patient presented with bilateral PE without any evidence of DVT or lower extremity venous insufficiency. We feel that if we had not considered MTS as a possible cause of her PE, she might have had another such life-threatening event in the future. Thus, it is of paramount importance to consider MTS as a possible cause in the correct clinical scenario.
